# Increased salivary oxytocin correlates with lower self-reported interoceptive accuracy in functional neurological disorders

**DOI:** 10.1016/j.ynstr.2025.100765

**Published:** 2025-10-19

**Authors:** Natascha Stoffel, Laure von der Weid, Josef Gross, Cristina Concetti, Rupert Bruckmaier, Selma Aybek

**Affiliations:** a Faculty of Science and Medicine, University of Fribourg, 1700, Fribourg, Switzerland; b Graduate School for Health Science (GHS), University of Bern, 3012, Bern, Switzerland; c Veterinary Physiology, Vetsuisse Faculty, University of Bern, 3012, Bern, Switzerland

**Keywords:** Conversion disorder, Interoception, Interoceptive accuracy scale (IAS)

## Abstract

**Introduction:**

Functional Neurological Disorder (FND) is shaped by psychosocial stress, early adversity, and neuroendocrine dysregulation. Oxytocin (OXT), a hormone central to stress regulation and interoception, remains largely unexplored in FND.

**Methods:**

In this cross-sectional study, salivary OXT was assessed at four timepoints across 87 participants (42 FND and 45 sex-age-matched healthy controls), including appetite and satiety and hormonal factors (intake of hormonal contraception or menstrual cycle phases) as covariates. Self-reported interoception, attachment style, childhood trauma and sexual functioning were assessed allowing analysis for association.

**Results:**

Patients with FND exhibited higher OXT concentrations (averaged across four timepoints: d = 0.55, p = 0.031), which remained when controlling for covariates. Appetite and satiety specifically modulated OXT levels at different timepoints, underlying the group difference after lunch (p = 0.006) and at the end of the study visit (p = 0.035). Self-reported interoceptive accuracy was negatively correlated with OXT (r = −0.31, p = 0.014) and insecure attachment was positively correlated with OXT in controls (r = 0.42, p = 0.005), but not in FND. No associations of OXT and childhood trauma or sexual functioning reports were found.

**Discussion:**

The elevated salivary OXT levels observed in patients with FND may reflect a dysregulated or compensatory neuroendocrine response. The combination of higher OXT and lower self-reported interoceptive accuracy suggests that OXT may be upregulated as an attempt to modulate bodily stress or restore homeostatic balance. The absent association of OXT with attachment style in FND specifically supports its role in dealing with socio-affiliative stress.

## Highlights

• Elevated salivary oxytocin in patients with functional neurological disorder.
• Oxytocin negatively correlates with self-reported interoceptive accuracy.
• Insecure attachment and oxytocin are associated in controls, but not in FND.
• Oxytocin as stress-buffer should be considered along interoceptive variables.
• Dysregulated oxytocinergic release might be related to FND's underlying stressors.

## Graphical abstract

**Figure:**
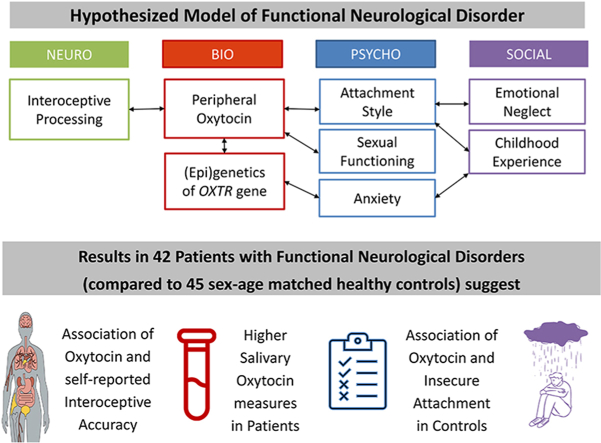

## 1 Introduction

Functional Neurological Disorder (FND) is a complex neurological condition characterized by a demonstration of motor, sensory, and cognitive symptoms from altered neuronal network activities. Historically, these such FND symptoms, such as tremor, dystonia, gait disorder or functional seizures, have been summarized under the term *Conversion Disorder*, following Sigmund Freud's theory of that repressed psychological stressors can convert into physical symptoms (Hallett et al., 2022). While different stress-related trigger factors are still widely recognized, this theory of a trauma necessity is outdated (Nicholson et al., 2016). Increasingly, it is recognized as a disorder with a multifactorial pathophysiology, encompassing biological, psychological, and social dimensions (Aybek and Perez, 2022). Among the recognized psychological and psychosocial contributors, early-life adversity and chronic stress have however still be identified as key risk factors for FND, but rather through acting through long-term dysregulation of the stress response system (Keynejad et al., 2019; Ludwig et al., 2018; Weber et al., 2023).

The hormone oxytocin (OXT) has gained attention in the last decade for its impact on human behavior and health (Carter et al., 2020). The broad involvement in reproductive behaviour, physiological functions, as well as social bonding has been widely discussed since decades for a potential use in pharmacology (Viero et al., 2010), making the oxytocinergic system also in recent advancements a key target to modulate key physiological processes, including autonomic regulation and stress response involved in neurological or psychiatric disorders (Jurek and Neumann, 2018; Young Kuchenbecker et al., 2021). OXT exerts regulatory effects on the hypothalamic–pituitary–adrenal (HPA) axis via neuroimmunological mechanisms (Danese and Baldwin, 2017; Engel et al., 2020; Li et al., 2017; Lindholm et al., 2020) and has been implicated in the mitigation of stress-related disorders such as anxiety and post-traumatic stress disorder (Engel et al., 2020; Nisbett et al., 2023; Onaka and Takayanagi, 2019; Yoon and Kim, 2020; Young Kuchenbecker et al., 2021). Importantly, alterations in the oxytocinergic system have been associated with adverse life experiences, including childhood maltreatment and insecure attachment styles or distrust (Bertsch et al., 2013; Buchheim et al., 2009; McQuaid et al., 2016; Müller et al., 2019). Taking in account the role of stress in FND, these findings suggest that OXT may represent a key hormonal mediator linking psychosocial vulnerability to the development or maintenance the disease (Keynejad et al., 2019; Paetzold et al., 2017; Weber et al., 2023; Williams et al., 2019).

Recent literature has highlighted the role of OXT in modulating interoception - the processing of internal bodily signals essential for maintaining homeostasis and allostasis (Liu et al., 2021; Quattrocki and Friston, 2014; Sabatier et al., 2013; Smith et al., 2022). Intranasal OXT administration enhanced the accuracy in performance on the traditional heartbeat counting tasks (HCT) and increased the heartbeat-evoked potential (HEP) amplitudes, a neuronal marker of encoding interoceptive cardiac signals with high precision (Zhou et al., 2024). This potential intersection between the oxytocinergic and interoceptive systems is of relevance for FND as well, a condition in which interoceptive dysfunction is increasingly recognized as a core feature (Elkommos et al., 2023; Flasbeck et al., 2024; Jungilligens and Perez, 2024; Pick et al., 2020; Ricciardi et al., 2021; Stoffel et al., 2025).

Interoceptive signalling could also be linked to the processing of hunger, satiety, and appetite which are central to the homeostatic monitoring, and OXT plays also a key role in this context. It not only modulates the perception of hunger and fullness but also influences neuroendocrine stress responses, emotional and reward-related processing of food, and broader homeostatic mechanisms (Liu et al., 2021; Onaka and Takayanagi, 2019). Moreover, OXT levels in blood significantly decrease in response to food intake in young healthy female adults (Gunesli et al., 2025) and are associated with perceived hunger and satiety following a meal (Aulinas et al., 2018). Early studies in rodents further found an increase of OXT after feeding but also aversive and nausea inducing agents (Verbalis et al., 1986), implicating the OXT system in further interoceptive signalling pathways. In fact, activation of the oxytocinergic system through administration of other related components can reduce the release of OXT to the bloodstream, while increasing OXT concentration in the brain, and with that modulating very specifically the feeling of appetite and body energy balance in the central nervous system (CNS), as well as physiologically (Sabatier et al., 2003). Therefore, when measuring OXT, the inclusion of such confounding variables is essential to ensure valid interpretation of OXT values and to acknowledge its involvement in interoceptive signalling.

In addition to its role in stress regulation and interoception, sexual functioning represents another relevant yet underexplored domain in the context of FND. Historically, the disorder has often been linked to sexual trauma, a view that has contributed to enduring stigma and oversimplified etiological models (Edwards and Aybek, 2020). While it is now widely recognized that this perspective is outdated and reductive, trauma—including sexual trauma—remains a significant risk factor for the development of FND in some individuals (Asadi-Pooya et al., 2021; Libbon et al., 2025). Importantly, sexual functioning itself is increasingly acknowledged as an integral component of overall physical and emotional well-being and deserves consideration within both clinical and research frameworks (Dekker et al., 2020; Fischer et al., 2022). OXT plays a key role in human sexual behaviour, modulating both sexual activation and satisfaction and emotional intimacy (Caruso et al., 2018; MacDonald, 2013; Salonia et al., 2005; Veening et al., 2015, 2015, 2015). Moreover, sexual arousal involves both emotional and physiological responses (e.g., feeling of desire, genital sensations, and affective touch) which are inherently interoceptive and correlated (Berenguer et al., 2019). Consequently, disruptions in interoceptive processing as discussed in FND, may also extend to domains of sexual functioning. Nonetheless, no study has so far investigated sexual functioning in FND in relation the interoceptive and oxytocinergic system.

Finally, the exploration of group differences in peripheral OXT for patients with FND are limited to only two studies. One did not identify a significant group difference in plasma OXT (Örnek et al., 2021), and the second, conducted by our group, investigated OXT levels in saliva yielding significant interaction with genetic and epigenetic measures specific to FND, but without an overall group difference in peripheral OXT (Weber et al., 2025). As none of the two studies has carefully controlled for key confounding factors such as appetite, satiety, and circadian rhythms, which can significantly influence OXT levels (Aulinas et al., 2018; Olszewski et al., 2016; Onaka and Takayanagi, 2019; Van Dam et al., 2018), we wanted to test in a new cohort, whether salivary OXT would differ when designing the study for the purpose of investigating the oxytocinergic and interoceptive system. The measurement in saliva was chosen as an easy and non-invasive collection method, which seems to better reflects central OXT concentrations compared to plasma measures (Leng and Sabatier, 2016; MacLean et al., 2019; Martin et al., 2018; McCullough et al., 2013). OXT can be released into the bloodstream via the pituitary, but also released to CNS regions via hypothalamic nuclei (Jurek and Neumann, 2018; Neumann, 2002), while the release from the CNS to saliva, as well as from plasma to saliva are discussed as potential routes of circulation (López-Arjona et al., 2023). Recent evidence also reports that manipulating OXT levels in the bloodstream does not alter salivary levels in dairy cows (Wellnitz et al., 2025), and that salivary OXT correlates better with OXT levels in cerebrospinal fluid (CSF) than plasma OXT does, in a human clinical population (Martin et al., 2018). Additionally, salivary OXT can reliably be modulated by activities known to trigger the oxytocinergic system at central level (e.g. changing the processing of information, such as known for OXT as a neurotransmitter), while not responding to activity focused on target organs, i.e. breastfeeding (Jong et al., 2015). Yet, other high OXT-releasing events, such as labour would also release higher levels of OXT in the central system (i.e. CSF), for it to benefit the adaptations required during birth as well as postpartum (Uvnäs-Moberg et al., 2019). These findings together suggest that salivary OXT may be related to OXT in CSF independently of the bloodstream, but further research is needed to uncover how OXT reaches the saliva from the central nervous system and how its salivary levels relate to its central actions. Further the use of saliva measurement easily allows four different timepoints, as suggested for higher overall reliability (Martins et al., 2020). Specifically, we assessed OXT concentrations at four timepoints across the day, carefully chosen to control for fluctuations related to food intake and satiety. We further integrated validated questionnaires on attachment style, childhood trauma and sexual functioning, allowing us to test for known psychosocial moderators of OXT function. To account for the potential interplay between oxytocinergic and interoceptive systems, we included self-report measures of interoception in our analyses.

**Fig. 1:**
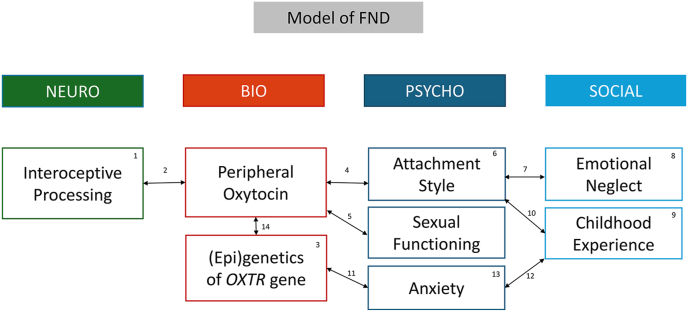
**Illustration of hypothezised disease model with involved concepts.** Please note that the model, as well as the references listed, are not complete. The asterix in the reference list indicate studies in FND populations. References: ^*1*^∗ Elkommos et al., 2023; Koreki et al., 2020; Ricciardi et al., 2021, 2016 | ^*2*^Quattrocki and Friston, 2014; Smith et al., 2022; Xin et al., 2020 | ^3^∗ Apazoglou et al., 2017 | ^4^Alaerts et al., 2019; Buchheim et al., 2009; Tops et al., 2007 | ^5^Caruso et al., 2018 | ^6^Cuoco et al., 2021; Holman et al., 2008; Jalilianhasanpour et al., 2019 | ^7^Kim et al., 2021; Müller et al., 2019 | ^8^∗ Weber et al., 2023 | ^9^∗ Ludwig et al., 2018 | ^10^∗ Williams et al., 2019 | ^11^Notzon et al., 2016 | ^12^Krause et al., 2018 | ^13^∗ Perez et al., 2020 | ^14^∗ Weber et al., 2025 |

In summary, the aim of our study was to generate a more reliable and nuanced understanding of the oxytocinergic system in FND and to explore for the first time its associations with psychosocial vulnerability, and interoceptive processes. Fig. 1 illustrates the concepts investigated here in a neuro-bio-psycho-social model of FND.

## 2 Methods

### 2.1 Participants

The study was carried out at the Cantonal Hospital of Fribourg (HFR) in the localities of the University of Fribourg and was part of a large study on altered interoception in patients with a total of N = 92 participants. For detailed discussion on the various interoceptive task, we kindly refer the reader to previous publications (Stoffel et al., 2025). Sample size estimation was based on prior data examining salivary cortisol in FND and HC (Weber et al., 2023), due to the similarities of the stress-related hormone and the lack of data on salivary OXT, with an estimation of f = 0.2 as an effect size. Using G∗Power (v3.1), for a mixed-design ANOVA (two groups, four time points) with α = 0.05 (two-tailed) and 95 % power, which lead to the requirement of N = 84 participants (42 per group) to detect a reliable group difference. Patients with FND (ICD-11/DSM-5: F44.4–44.7), aged >18 and capable of judgment, were included. Healthy controls (HCs) were recruited via flyers, word-of-mouth, and online advertisements. Exclusion criteria for both groups comprised: (a) major depression, psychosis, or other severe comorbidites, (b) brain surgery or medical implants, (c) substance abuse, (d) cardiac disorders, and (e) pregnancy/breastfeeding (verified via urine test for females of childbearing age). The study was approved by the local Ethics Committee of the Canton Bern (2023-00469), registered at clinicaltrial.gov (NCT06084325), and conducted according to the Declaration of Helsinki. All participants provided informed consent.

### 2.2 Psychometric assessment

**Fig. 2:**
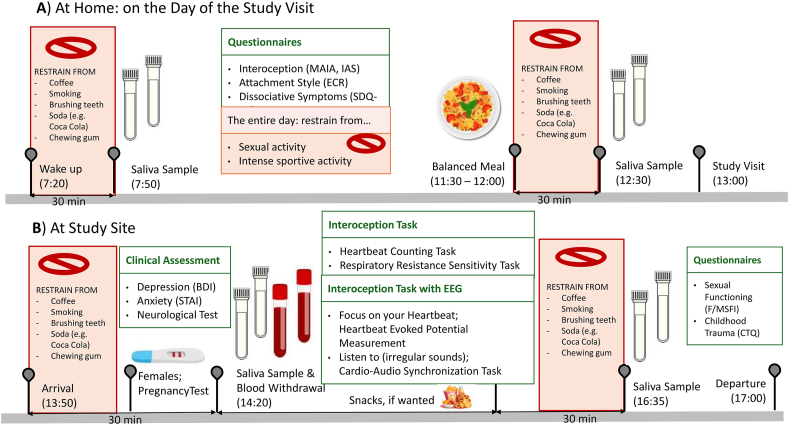
**Illustration of Study Design.** Part A) was at home and done independently, part B) was on site at the Cantonal hospital of Fribourg (HFR). Please note that some variables that were assessed in the study, are not content of discussion in this manuscript (e.g. blood withdrawal that served the purpose of analysing of genetic and epigenetic alterations of the *OXTR* and other stress-related genes, or outcomes of EEG measurements that are discussed in other publications).

All participants underwent a battery of tasks and measurement to assess interoceptive processing and related concepts. The study design is illustrated in Fig. 2.

Participants completed a series of validated questionnaires including the Somatoform Dissociation Questionnaire (SDQ-20; Nijenhuis et al., 1996), the Beck's Depression Inventory (BDI-II; Beck et al., 1996), the State-Trait Anxiety Inventory (STAI; Spielberger et al., 1970). To assess interoceptive self-report, participants completed the Multidimensional Assessment of Interoceptive Awareness (MAIA; Mehling et al., 2012). and the Interoceptive Accuracy Scale (IAS; Murphy et al., 2020). Also, as a self-reported assessment of attachment style using the Experience of Close Relationship was completed (ECR-R; Fraley et al., 2000), along with the Childhood Trauma Questionnaire (CTQ; Bernstein et al., 2003) and the Female/Male Sexual Functioning Index, dependent on self-reported sex (FSFI and MSFI respectively; Kalmbach et al., 2015; Rosen et al., 2000). We collected clinical information regarding medication, comorbid diagnoses, and for female participants, use of hormonal contraception, menopause or menstrual cycle phases. For latter, phases were divided into menstruation, follicular, ovulation, luteal phase; details on assessment in Supplementary Material. Patients also underwent a neurological examination including the clinical global impression (CGI; Guy, 1976), and the short Functional Movement Disorder Scale (SFMDRS; Nielsen et al., 2017).

For the assessment of interoceptive accuracy, the traditional cardiac heartbeat counting task (HCT), and the novel respiratory resistance sensitivity task (RRST) were implemented. For detailed discussion on both tasks, we kindly refer to previous publications (Stoffel et al., 2025).

### 2.3 Salivary oxytocin

Salivary OXT was assessed at four time points per participant. Fig. 2 clarifies how these time points were integrated in the study. For the part at home (Fig. 2A) all participants were instructed in advance via telephone and written manuals on how to collect the first two samples independently at home. They were also told to restrain from sportive or sexual activity on the day of the study visit, to control for the increase of OXT related to it (Hew-Butler et al., 2008; Jong et al., 2015). Participants collected the first sample 30 min after waking (mean time = 7:50 a.m., sd = 81 min), and the second 30 min after completing lunch (mean time = 12:26 a.m., sd = 45 min). We encouraged participants to have a mixed and balanced meal for lunch, with an example of a plate of rice and vegetables. The third and fourth samples were collected on-site: one before the start of interoceptive measurements (mean time = 2:18 p.m., sd = 42 min) and the one after the completion of all tasks at the end of the study visit (mean time = 4:35 p.m., sd = 60 min).

At each time point, two saliva samples were collected using two Salivette® tubes with cotton swabs (Sarstedt, Germany) accompanied by self-reports of satiety and appetite on a scale from 1 to 5. Following the final on-site collection, all eight tubes (two per time point) were centrifuged at 3000 rpm for 5 min. Pooling of the two samples from the same time point ensured sufficient volume for subsequent analysis. Samples were stored at −20 °C until batch analysis. Salivary OXT concentrations were quantified using ultra-sensitive enzyme-linked immunosorbent assay (ELISA) kit with a sensitivity of 15 pg/mL OXT (https://www.enzolifesciences.com/ADI-901-153A/oxytocin-elisa-kit/). Prior to individual sample analysis, the ELISA kit was validated using pooled saliva samples spiked with OXT at concentrations of 10, 20, 50, and 100 pg/mL. Recovery rates ranged from 110 % to 120 %, consistent with kit specifications. Due to the low native concentrations of salivary OXT, all samples underwent a 4-fold concentration step by extracting 1 mL of saliva and reconstituting in 250 μL of assay buffer.

### 2.4 Variable Manipulation

For OXT_average_ we averaged all available OXT measures per participant (even if a participant might have a missing value in one timepoint due to too little volume of saliva, which was the case for a total of N = 5 participants that each had one out of four timepoints missing).

Further, OXT levels are known to be higher in pre-menopause compared to post-menopause (Maestrini et al., 2018), during the ovulation phase compared to the luteal phase (Engel et al., 2019; Salonia et al., 2005), and participants with intake of hormonal contraception (Silber et al., 1987). Consequently, a flag variable of menstrual cycle was created to mirror the enhanced level of OXT by labeling all participants with the intake of hormonal contraception (yes vs no), and those who were currently in their ovulational phase.

For the high correlation of BDI-II depression and STAI-T anxiety scores (r = 0.78, p < 0.001), a sum score was calculated representing the affective symptom severity. Finally, sexual functioning and attachment style questionnaire scores were adjusted for comparability, with details described in Supplementary material.

### 2.5 Statistical analyses

Statistical analyses were performed using *R* software (version 4.3.3). Data were tested for normality using Shapiro-Wilk's test. Normally distributed demographic/clinical data were analyzed using two-sample *t*-test or Pearson correlation, non-normally distributed data using Wilcoxon rank sum test or Spearman correlation. Alpha-level for significance was set at *p* < 0.05.

Wilcoxon tests were calculated for average group difference in OXT. P-values and the effect size of Cohen's d for the analysis were reported, and where necessary control for multiple comparison using False Discovery Rate (FDR) was applied. Linear regressions were performed on OXT_average_ adding co-variates of no interest (sex and age, psychotropic medication, affective symptom score, or the menstrual cycle flag variable). Also, a linear mixed model was used to investigate the factor group (FND vs HC) and the factor of timepoint (waking, lunch, pretask, posttask) on the OXT values, accounting for the individual intercept in the repeated-measure setting. Satiety and appetite perception of the participant per timepoint were tested as interoceptive covariates in linear regressions, separate per timepoint. For association with other variables (such as childhood trauma or attachment style) linear regressions were analyzed with an interaction term, followed by a post hoc analysis to estimate the slope separately within each group. For each comparison, β respectively *b* estimates, and p-values are reported, along with the F-statistics of the full model, respectively the 95 confidence intervals (CI).

## 3 Results

### 3.1 Baseline characteristics

From our total sample of N = 92, two participants did not produce enough saliva to measure any OXT measurements and thus were excluded. Also, three statistical outliers were detected (75 % or 25 % Quartile ± 1.5∗IQR) and excluded for further analysis (with average OXT of 19.15, 22.13 and 0.00 pg/ml). Our final sample size thus consisted of N = 87 (FND: N = 42, mean age of 38.81 (sd = 11.87) and 71 % female, and HC: N = 45, mean age of 37.98 (sd = 13.34) and 71 % female. No differences in sex or age were found between groups, confirming a well-matched sample, and large enough according to the above-mentioned calculated sample size.

Patients with FND showed to score higher in depression, state and trait anxiety, somatoform dissociation and had a higher intake of psychotropic medication (p < 0.001) than controls. There was no difference in menopause, or those with a natural cycle to be in ovulation phase (p > 0.5), but patients with FND showed a higher percentage of intake of hormonal contraception (36.7 % compared to HC = 9.4 %, p = 0.015). Patients with FND reported lower on both interoception self-reports (parametric *t*-test for MAIA: p < 0.001, non-parametric Wilcoxon test for IAS: p = 0.026). While there was no difference in terms of attachment style (both non-parametric Wilcoxon test for ECR avoidant and anxious p > 0.6), patients with FND reported a higher score in experience of childhood trauma (non-parametric Wilcoxon test of p = 0.008). There was no overall group difference in sexual functioning (non-parametric Wilcoxon test of p = 0.072), but the subscales of desire, lubrication and orgasm was found to be lower in female patients with FND (FDR adjusted p = 0.045). Table 1.

**Table 1:** Demographics.

Variable	OverallN = 87	HC N = 45	FND N = 42	p-value
Female sex (count, %)	62.0 (71.3)	32.0 (71.1)	30.0 (71.4)	>0.9
Female Menstrual Cylce Hormones, N = 62				
*In menopause (count, %)*	*11.0 (17.7)*	*7.0 (21.9)*	*4.0 (13.3)*	*0.5*
*Intake of hormonal contraception (count, %)*	*14.0 (22.6)*	*3.0 (9.4)*	*11.0 (36.7)*	*0.015∗*
*In self-reported Ovulation Phase (count, %)*	*12.0 (34.3)*	*7.0 (31.8)*	*5.0 (38.5)*	*0.7*
Age (mean, SD)	38.4 (12.6)	38.0 (13.3)	38.8 (11.9)	0.8
Intake of psychotropic medication (count, %)	21.0 (24.7)	2.0 (4.7)	19.0 (45.2)	<0.001∗∗∗
Depression: BDI-II (median, IQR)	8.0 (12.0)	4.0 (7.0)	15.0 (13.8)	<0.001∗∗∗
State Anxiety: STAI-S (median, IQR)	32.0 (15.0)	28.0 (10.0)	39.0 (19.8)	<0.001∗∗∗
Trait Anxiety: STAI-T (mean, SD)	41.1 (11.7)	36.6 (9.7)	45.9 (11.9)	<0.001∗∗∗
Somatoform Dissociation: SDQ-20 (median, IQR)	27.0 (14.0)	23.0 (5.0)	36.0 (16.8)	<0.001∗∗∗
Self Report Interoceptive Awareness: MAIA (mean, SD)	22.4 (5.8)	24.6 (4.1)	20.0 (6.4)	<0.001∗∗∗
Self Report Interoceptive Accuracy: IAS (median, IQR)	85.0 (17.5)	89.0 (13.0)	80.0 (23.0)	0.026∗
Childhood Trauma: CTQ (median, IQR)	37.0 (26.0)	34.0 (15.0)	46.5 (27.8)	0.008∗∗
*Subscales*	*p-value (unadjusted)*		*p-value (FDR adjusted)*	

*Sexual Abuse*	*0.047∗*		*0.071*	
*Physical Neglegt*	*0.245*		*0.294*	
*Physical Abuse*	*0.006∗∗*		*0.037∗*	
*Emotional Neglect*	*0.043∗*		*0.071*	
*Emotional Abuse*	*0.033∗*		*0.071*	
*Minimization*	*0.426*		*0.429*	
Anxious Attachment: ECR (median, IQR)	2.4 (1.8)	2.6 (1.4)	2.4 (1.8)	0.7
Avoidant Attachment: ECR (median, IQR)	2.4 (1.6)	2.3 (1.2)	2.6 (2.1)	0.6
Sexual Functioning: FSFI/MSFI (median, IQR)	25.4 (12.0)	26.0 (8.0)	22.3 (20.2)	0.072
*FEMALE (FSFI), N = 62*	*p-value (unadjusted)*		*p-value (FDR adjusted)*	

*Satisfaction*	*0.272*		*0.272*	
*Pain*	*0.157*		*0.188*	
*Orgasm*	*0.017∗*		*0.045∗*	
*Lubcrication*	*0.012∗*		*0.045∗*	
*Desire*	*0.023∗*		*0.045∗*	
*Arousal*	*0.090*		*0.135*	
*MALE (MSFI), N = 25*	*p-value (unadjusted)*		*p-value (FDR adjusted)*	

*Satisfaction*	*0.035∗*		*0.862*	
*Orgasm*	*0.027∗*		*0.136*	
*Erection*	*0.952*		*0.952*	
*Desire*	*0.866*		*0.952*	
*Arousal*	*0.912*		*0.952*	

Note: Mean and sd is shown for normally distributed, and tested with a *t*-test. Median and IQR is shown for non-normally distributed data, and tested with a Wilcoxon-test. Count and percentage is shown for dichotomous data, and tested with a fisher test. Significance level is shown for p < 0.05 with ∗, p < 0.01 with ∗∗ and p < 0.001 with ∗∗∗. Note: For analysis on menstrual cycle hormones in N = 62 females only, ovulational phase was suggested with a reference of days 10–19 of natural cycle, in case they would not know based on tracking their cycle.

### 3.2 Group difference in oxytocin and over timecourse

**Fig. 3:**
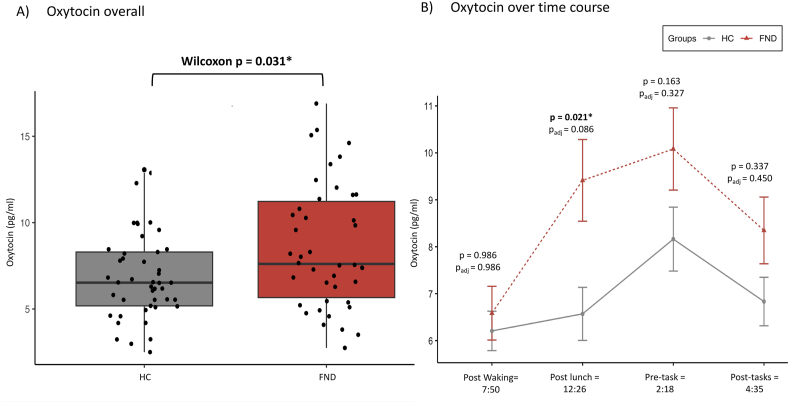
**Oxytocin average and over the time course, separately per group**. A) Overall Oxytocin scores in pg/ml per group, averaged across all timepoints per person, and using a Wilcoxon test due to the non-normal distribution of the values. B) Oxytocin values separate for the four time points assessed; the lines represent the mean scores with the standard error portrayed as bars. Timepoint waking is for 30mis following wake-up, lunch 30min following the completion of lunch, while pre- and posttask was collected before and after the study tasks which included interoceptive measurements. Below the timepoints the mean time overall is indicated. P-values represent the difference between groups, using again Wilcoxon tests with the p_adj_ representing adjusted p-value for FDR correction for multiple comparison. For N_waking_ = 43 FND and 46 HC, N_lunch_ = 42 FND and 47 HC, N_pretask_ = 42 FND and 46 HC and N_posttask_ = 42 FND and 47 HC.

Patients with FND had a higher OXT_average_ value, with a median of 7.61 pg/ml (IQR = 5.57) compared to controls with 6.52 pg/ml (IQR = 3.12); using a non-parametric Wilcoxon test: d = 0.55, p = 0.031. Fig. 3A. This group difference survived for the control of co-variates sex and age, affective symptoms, or flag variable of menstrual cycle flag (p < 0.041), but not when controlling for psychotropic medication (p = 0.067).

Running a linear mixed model including timepoint as a variable for repeated measures, we found both an overall effect of group and timepoint on OXT levels: Patients with FND demonstrated higher OXT values compared to HCs (*F*(1, 85.17) = 6.57, *p* = 0.012) while OXT also varied across the day (*F*(3, 254.12) = 8.57, *p* < 0.001). Specifically, OXT increased for FND from waking to lunch (β = 1.60, *p* = 0.003), from lunch to pretask (β = 2.71, *p* < 0.001), and from pretask to posttask (β = 1.21, *p* = 0.025). However, when running non-parametric Wilcoxon tests to test the group differences separately per time point, none of the p-values remained significant after correction for multiple comparison using FDR: *p* < 0.086) Fig. 3B.

### 3.3 Control for interoceptive covariates: satiety & appetite

Including for each timepoint the satiety and appetite rated as co-variates, we identified a group difference for the timepoint after lunch (p = 0.006) and at the end of the task (p = 0.035), while appetite was associated with the OXT after waking and post task (p = 0.040), and satiety was associated with the OXT at lunch (p = 0.033). Detailed results in Supplementary material, Table S1.

### 3.4 Oxytocin and interoception

We identified a negative correlation of the interoceptive self-report scores using the IAS questionnaire and the OXT_average_ (spearman correlation r = −0.31, p_adj_ = 0.014). Testing separately per time point, it was the time pretask (spearman correlation r = −0.34, p_adj_ = 0.006) that remained significantly associated with the IAS. No other interoceptive variable was correlated with the OXT_average_ scores; neither the multidimensional total score of the MAIA, nor any subscale when testing them separately, and also none of the behavioral markers of respiratory or cardiac accuracy collected within the study (Stoffel et al., 2025).

### 3.5 Oxytocin and risk-factors: childhood trauma and attachment style

**Fig. 4:**
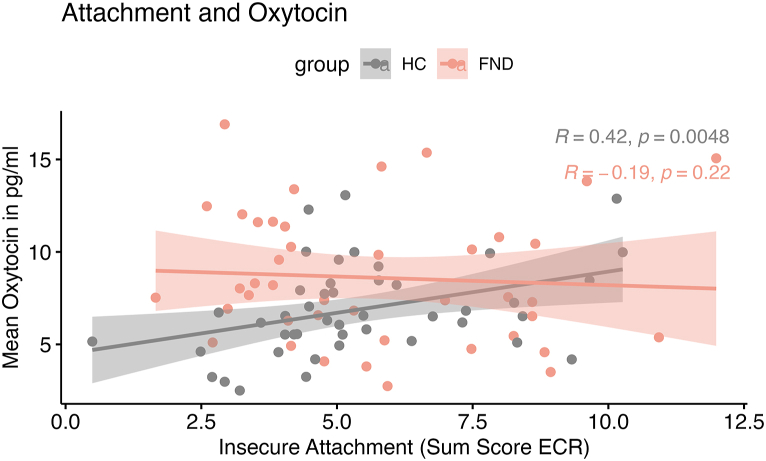
**Group-specific slopes on self-reported Insecure Attachment and Mean Oxytocin Levels.** We report here Spearman correlation and illustrate the trend for interaction (• = −0.54, p = 0.063), next to the two main effects of group and attachment insecurity. Patients with FND in red do not show a correlation of OXT and the insecure attachment score, which was derived as the sum of the self-reported subscales suggesting either avoidant or anxious attachment style using the Experience of Close Relationship questionnaire (ECR-R), while for HC in grey, there is a positive association.

Modeling the involvement of hypothesized risk-factors, we identified both a main effect of group (β = 4.66, p = 0.008) and insecure attachment (β = 0.45, p = 0.041), along with a tendency for an interaction (β = −0.54, p = 0.063) with OXT_average_ (F(3,83) = 3.80, p = 0.013). A post-hoc analysis indicated a slope of *b* = 0.45 (95 % CI [0.02, 0.87]) as a significant positive association for the HC group, while this association was missing in patients with FND (*b* = −0.09, CI [−0.47, 0.28]). Fig. 4. Supplementary Fig. S1 further shows the regression lines, separate per insecure dimension (anxious and avoidant attachment). For the experience of childhood trauma, no interaction was detected with group to predict OXT_average_, and no correlations were identified when testing the individual subscales for patients with FND (Wilcoxon p > 0.097). Also, no correlations to symptom severity have been identified with OXT_average_ in the patient group.

### 3.6 Oxytocin and sexual functioning

While some subscales of the female sexual functioning index were lower for patients with FND, namely orgasm, lubrication and desire (Wilcoxon p_adj_ = 0.045), the overall scores were not different between groups. Neither the overall score, nor the identified subscores were correlated with OXT_average_. Sexual functioning and group did neither show a main effect, nor an interaction on the overall peripheral values of OXT_average_, both when testing it separately per sex, or overall.

## 4 Discussion

### 4.1 Higher OXT in FND

Our findings demonstrate a significant group difference in salivary OXT_average_ levels between patients with FND and HCs, an effect surviving also the inclusion of co-variates of no interest such as sex, age and cycle related alterations in OXT. Previous literature in FND suggested no difference between patients and HCs in plasma whereas both low sample size but also the methodological difference of extraction might be the reason for this inconsistency (Örnek et al., 2021). However, in a previous publication of our group (with a different, but large study population) we also found no group difference in saliva (Weber et al., 2025). The key difference is that our current study was carefully designed to measure OXT, combining different timepoints per participants (i.e. four measurements as required for higher validity; (Martins et al., 2020), and assessing potential co-variates or ask to restrain from known confounding activities (e.g. sexual or sportive activity (Hew-Butler et al., 2008; Jong et al., 2015);) before the study visit. This leads to the conclusion, that our current work and results represent a higher validity concerning the baseline salivary OXT in the studied populations. The increased salivary OXT levels support previous evidence of enhanced salivary levels associated with depressive symptoms in adults (Holt-Lunstad et al., 2011), as well as with the finding of enhanced values for adolescents admitted to a psychiatric ward compared to HCs (Gizuterman et al., 2025). Yet, a study with healthy adults during the COVID-19 pandemic demonstrated that salivary OXT was lower in those individuals reporting higher physical pain and stress, which underlines the interaction with stressful experience in a still healthy population in the different direction (Schneider et al., 2023). Thus, there might be other (e.g. genetic, or epigenetic) factors at play for patients, or there might be a pattern of overcompensation over time. In other words, there could be an increase of OXT release after prolonged stress-induced reduction, similar to the reduction of cortisol after a continued enhancement of cortisol values through prolonged stress (Fries et al., 2005). This hypothesis is further supported by the finding that an acute increase of OXT via intranasal administration would lead to a higher level of trauma-related intrusive memories in healthy women (Schultebraucks et al., 2022). However, as our group difference would remain for the control of sex, age, affective symptoms, and menstrual cycle related covariates, but not medication, we cannot neglect the effect of medication on the altered oxytocinergic system in our patient group. This warrants consideration for further research, as the increase of OXT might be linked to the intake of psychotropic medication that should be discussed for patients before prescribing them, as well as considering the oxytocinergic system itself as an alternative candidate for pharmaceutical treatment (Marazziti et al., 2022).

### 4.2 Higher OXT depending on timepoint

In our sample, the disparity between group is most evident after lunchtime, even though the non-parametric Wilcoxon test would not survive the threshold for significance after controlling for multiple testing. However, at this timepoint, satiety ratings were associated with the OXT levels, indicating that postprandial interoceptive states may be correlated to OXT secretion. This aligns with previous research identifying satiety and appetite as relevant covariates of OXT (Aulinas et al., 2018), and supports its proposed role in regulating the perception of internal bodily states during food intake, which are key interoceptive signals (Liu et al., 2021). There is existing evidence that OXT is involved in the regulation of eating behaviour and energy balance, particularly in response to nutrient intake and caloric homeostasis (Aulinas et al., 2018; Liu et al., 2021; Onaka and Takayanagi, 2019). While it is expected to see and increase of OXT through the day (Gizuterman et al., 2025), the group difference in OXT was also particularly significant at the end of the study visit (but again not statistical different using non-parametric tests corrected for multiple comparison). Thogether, these findings still point towards the interpretation, that FND-related OXT differences is more pronounced after high (i.e. after lunch) and low satiety (i.e. end of study visit). These specific timepoints may reflect a context-sensitive oxytocinergic system, where physiological states such as hunger and satiety dynamically modulate neuroendocrine responses. Importantly, adjusting for these factors not only clarified group effects but also strengthened the interpretation of OXT elevations in FND as potentially dysregulated in relation to interoceptive signals, rather than purely baseline dependent.

### 4.3 Higher OXT correlated with lower interoceptive accuracy

The negative correlation of interoceptive accuracy self-report indicates that individuals, who reported lower accuracy in perceiving their internal bodily signals, demonstrate also higher OXT concentrations. These findings suggest a relationship between OXT and interoception in FND highlighting its role in modulating interoceptive processes (Ainley et al., 2016; Quattrocki and Friston, 2014). This relationship was independent of group and specific to the IAS, targeting the self-reported accuracy. We did not find such a correlation when using the MAIA, assessing the multidimensional awareness of interoceptive signals, nor when using behavioural measures of cardiac or respiratory accuracy. Thus, supporting the proposed model of OXT modulating self-reported interoceptive precision, rather than general awareness (Ainley et al., 2016; Quattrocki and Friston, 2014). In fact, the combination of elevated OXT in FND and its inverse relationship with satiety, appetite, and interoceptive accuracy may reflect a compensatory or dysregulated neuroendocrine response—potentially aimed at dampening distress regarding internal bodily states. In this context, OXT might act to blunt the salience of interoceptive cues such as hunger or satiety, contributing to an atypical bodily experience that might be part of the characteristics of FND.

### 4.4 No association with childhood trauma

Although the FND group reported significantly higher levels of childhood trauma, we found no correlation between OXT and total trauma scores or subscales, nor an interaction with group. While OXT has been discussed to be able mitigate stress-related disorders, feelings of anxiety or the severity of post-traumatic stress disorder (Engel et al., 2020; Onaka and Takayanagi, 2019; Yoon and Kim, 2020; Young Kuchenbecker et al., 2021), we did not find an association between the questionnaire score and salivary measurements. In fact, the previous study assessing plasma OXT in FND did also not find a statistical difference of OXT in patients, but discussed a non-statistical trend for lower OXT values given the presence of history of childhood trauma (Örnek et al., 2021). Together, our findings suggest, that early adverse experiences may not directly influence OXT concentrations in a way that differs between FND and HCs, or that other factors such as epigenetic and genetic variation might play a key modulating role (Chen et al., 2020; Lee et al., 2022; Notzon et al., 2016; Weber et al., 2025).

### 4.5 Lack of attachment-dependent OXT values in FND

In contrast, attachment style showed a differential association with OXT depending on group. Among HCs, insecure attachment was positively correlated with higher OXT levels, whereas no such relationship was observed in the FND group. Notably, the overall level of attachment insecurity did not differ between groups, indicating that the interaction effect reflects a difference in how attachment relates to OXT across diagnostic status. In HCs, elevated OXT in individuals with insecure attachment may represent a compensatory upregulation of oxytocinergic signalling, potentially aimed at enhancing affiliative behaviour or mitigating stress (Bartz, 2016; Crockford et al., 2018), and similar to the findings of adolescents in a psychiatric ward (Gizuterman et al., 2025). This interpretation aligns with prior research demonstrating that intranasal OXT promotes more secure attachment responses (Buchheim et al., 2009), reducing excessive amygdala-hippocampal connectivity that is associated with emotional dysregulation (Alaerts et al., 2019) and that an intervention of warm touch by a married partner has shown to decrease the enhanced OXT levels in depressed patients (Holt-Lunstad et al., 2011). The absence of this compensatory association in the FND group may reflect a dysfunction within the oxytocinergic system, wherein OXT would fail to fulfil its modulatory role in attachment and stress regulation, contributing to the altered socio-affective and interoceptive processing observed in FND (Young Kuchenbecker et al., 2021).

### 4.6 No involvement of sexual functioning in oxytocinergic associations

Finally, sexual functioning has largely been neglected in contemporary FND research. In our cohort, we identified impairments in specific subscales of sexual functioning among female FND patients (i.e., orgasm, desire, lubrication), but no overall group difference. Furthermore, OXT concentrations were not associated with sexual functioning in either group or sex (neither total nor subscores), even though OXT is known to play a role in sexual behaviour including satisfaction, and emotional intimacy (Caruso et al., 2018; MacDonald, 2013; Salonia et al., 2005; Veening et al., 2015, 2015, 2015). These null findings in our study, contradicting to previous literature, may be attributed in part to methodological differences, particularly the use of salivary rather than plasma OXT measurements (Caruso et al., 2018). Alternatively, the absence of an association indicates that OXT dysregulation in FND is not related to sexual functioning, or that the experience of sexual functioning may be context-dependent and modulated by additional factors such as relationship quality, psychological distress, or hormonal status (considering the group differences in contraceptive intake, interoceptive accuracy, and correlation to attachment style).

### 4.7 What we may measure with salivary OXT

Related to all this, we further want to discuss the methodological approaches and the interpretation of salivary measurement. The non-invasive sampling of saliva OXT determination is an important advantage for clinical studies. However, it appears that saliary OXT is rather a parameter on its own instead of just reflecting OXT concentration in peripheral cirtulation. The biological effects of peripheral plasma OXT are mainly related to reproductive organs including mammary gland which is obiously not necessarily reflected by changes of salivary OXT (Jong et al., 2015; Martins et al., 2020). Martin et al. (2018) concluded that salivary OXT may rather represent the concentration associated with the neuropeptide in the brain than in peripheral circulation. A recent study conducted in lactating cows confirmed increased blood plasma OXT concentrations in response to mammary gland stimulation in the absence of any concomitant changes of salivary OXT concentration (Wellnitz et al., 2025). In addition, the intravenous injection of OXT up to high supraphysiological dosages did not alter salivary OXT concentrations (Wellnitz et al., 2025). Similarly, while social stress, self-induced running and sexual activity leads to a change in salivary OXT in humans, OXT would not spike in response to breastfeeding either (Jong et al., 2015). In conclusion, salivary OXT does obviously not reflect OXT in peripheral blood circulation, it remains unknown if and why salivary OXT can reflect OXT activity in the CNS.

### 4.8 Limitations

While we tried to control the salivary collection to specific time points, standardizing the sampling to crucial time points, there still might be variability from individual adherence to protocol, but also individual differences. Some might have woken up very early, or have eaten lunch very early, while others tend to do that later. Also, we could not control the intake of the lunch (what, when and how much was eaten), we just encouraged them to have a well-balanced meal that was finished 30min before the second saliva sampling. The difference in daily routine though might have influenced this a lot. Also, while we instructed individuals to restrain from sportive and sexual activities on the day of the study visits, we would not know whether they would stick to this protocol, or whether OXT values might still be increased in some individuals based sportive or sexual activities the night before. Finally, knowing that OXT is a stress-related buffer hormone, the fact that they would come to the hospital to do the study visit might have influenced the OXT release, and maybe so specifically for patients.

Related to the OXT measurement, it must be highlighted that we do not fully know how salivary measurement might represent the OXT in the brain, such as the availability of OXT as a neurotransmitter in the central system. Our methods do not allow on any interpretations on that, hence further investigation needs to shed more light on what exactly is represented by salivary OXT, and how this might mirror neuronal processing.

A lot of our reported results are based on self-report questionnaires, which may not fully capture the situation objectively (in particular for attachment style and sexual functioning). While we want to stress that the self-report represent the subjects experience, there might also be a bias involved in the retrospective evaluation or perception and may be influenced by cognitive or emotional biases, particularly in populations with altered body awareness or emotional processing (Handy and Meston, 2016).

While controlled for menstrual related fluctuation of OXT using self-report, this might not be as accurate as using hormonal markers to classify phase, or as comparable by strictuly limiting study participation to a specific phase in natural cycling women only.

Another limitation is that we here do not have genetic or epigenetic variables allowing to assess the interaction of peripheral OXT on the oxytocin receptor gene (*OXTR*) which has previously been seen to interact (Notzon et al., 2016; Weber et al., 2025). Future studies and analysis should take that into account, for obtaining a more comprehensive understanding of the oxytocinergic system.

## 5 Conclusion

In summary, our findings offer novel insights into the role of the oxytocinergic system in FND by presenting results from a carefully designed study and thus being able to account for known covariates such as appetite and satiety, menstrual cycle and hormonal contraception, and various timing through the day. We were able to reveal elevated salivary OXT levels and altered associations with interoceptive and socio-affective processes in FND. The observed OXT elevations, particularly after lunch and following interoceptive tasks, are consistent with theoretical models positing a role for OXT in homeostatic regulation. Across groups, higher OXT was associated with lower self-reported interoceptive accuracy, suggesting a potential compensatory or dysregulated neuroendocrine response aimed at mitigating uncertainty or distress linked to disrupted bodily signalling. While no associations were found between OXT and childhood trauma, a group-specific dissociation emerged in relation to attachment: in HCs, insecure attachment correlated with higher OXT levels—supporting a compensatory role in socio-affective regulation—whereas this association was absent in FND, pointing to a disruption of oxytocin's modulatory function regarding its socio-affiliative effect. Together, these findings position salivary OXT as a candidate biological marker of vulnerability in FND, particularly in relation to interoceptive and attachment-related dysfunctions and support the integration of oxytocinergic mechanisms into future models of FND pathophysiology and intervention strategies.

## CRediT authorship contribution statement

**Natascha Stoffel:** Writing – review & editing, Writing – original draft, Visualization, Project administration, Methodology, Investigation, Formal analysis, Data curation, Conceptualization. **Laure von der Weid:** Writing – review & editing, Investigation. **Josef Gross:** Writing – review & editing, Validation. **Cristina Concetti:** Writing – review & editing. **Rupert Bruckmaier:** Writing – review & editing, Validation. **Selma Aybek:** Writing – review & editing, Funding acquisition.

## Funding

This work was supported by the Swiss 10.13039/501100001321National Research Foundation Professorship Grant PP00P3_176985.

## Declaration of competing interest

The authors declare that they have no known competing financial interests or personal relationships that could have appeared to influence the work reported in this paper.

## Data Availability

The code and the data, from those participants who constented for further use of their data, is made public at following github respository: https://github.com/FND-ResearchGroup/OXT_FND_NS.
