# Supplementary material for: Increased salivary oxytocin correlates with lower self-reported interoceptive accuracy in functional neurological disorders
Source: Neurobiol Stress. 2025 Oct 19;39:100765. doi: 10.1016/j.ynstr.2025.100765 (PMC12589955; doi:10.1016/j.ynstr.2025.100765)
Supplement: Multimedia component 1 [file mmc1.docx]

**Increased salivary Oxytocin correlates with
lower self-reported Interoceptive Accuracy in
Functional Neurological Disorders**

Natascha Stoffel^1,2^, Laure von der Weid^1^, Josef Gross^3^,
Cristina Concetti^1^, Rupert Bruckmaier^3^, Selma Aybek^1^

**Author affiliations:**

1 Faculty of Science and Medicine, University of Fribourg, 1700 Fribourg, Switzerland

2 Graduate School for Health Science (GHS), University of Bern, 3013 Bern, Switzerland

3 Veterinary Physiology, Vetsuisse Faculty, University of Bern, 3012 Bern, Switzerland

**Information on Menstrual Cycle**

We here provide the exact questionnaires that were used to assess menstrual cycle information for females only:

Please answer the following questions about your menstrual cycle. If you track your cycle in a calendar/app, you are of course welcome to use this guide to help you answer the questions. However, please also answer the questions as accurately and precisely as possible. Feel free to use the comments box to give us more information, or to share any concerns you have about the answer you gave. Thank you very much.

Do you use hormonal contraception or other hormonal preparations, e.g. for menopausal symptoms?

- Yes
  If yes: Please indicate the type and method of hormonal contraception/ hormonal preparations (e.g. (mini)pill, nuvaring, hormonal IUD, etc.) _________
- No

Do you currently have a natural menstrual cycle?

- Yes

If yes: Do you have a (more or less) regular cycle?

- - Date of last period ? _________
  - Duration of the cycle (normally) plus minus a few days: _________
  - In which phase of your cycle are you currently in ?
    - Currently menstruating (menstrual blood coming out, for our study, we'll use day 1-4 of cycle as reference)
    - Follicular phase (phase PRIOR ovulation; for our study, we'll use day 5-9 of cycle as reference)
    - Ovulation phase (phase AROUND ovulation, can be noticed by a pulling sensation at the side, for example. For our study, we'll use day 10-19 of cylce as reference)
    - Luteal phase (phase AFTER ovulation, can be noticed for example by psychological and physical premenstrual symptoms (PMS) : For our study, we'll use day 19-30 of cycle as reference.

NOTE: If you do not know which stage you are in, please select the answer that is most likely to be the case

- No

If no: Are you in menopause?

- - Yes

If yes: How long have you been in menopause? _________

- - No

If no: Other reason for missing menstrual cycle? _________

**Variable Manipulation: Sexual Functioning and Attachment Style**

As sexual functioning had been reported previously to affect OXT values,(38) we assessed it in a sex-specific manner using the female or male version of the sexual functioning index (FSFI or MSFI respectively) according to their self-reported sex and gender. As the FSFI has a different structure and number of subscale (FSFI ranging from min 2- max 36 and MSFI ranging from min 2-max 30), the MSFI was rescaled to also fit to the FSFI variable structure, and they were combined as a common variable of sexual functioning (ranging from 2-36).

At the end of completing the ECR-R questionnaire on attachment style, participants indicated the type of relationship they had in mind while responding (romantic: experienced, romantic: imagined, friendship, or family). To account for potential variance in attachment scores due to differences in relationship context, we fitted separate linear models for each ECR-R subscale (avoidant and anxious), using the reported relationship type as a predictor. The residuals from these models were then added to the overall sample mean of each respective subscale, yielding adjusted scores that control for relationship-type-related bias per participant.

**Control for Interoceptive Covariates: Satiety & Appetite**

We ran linear regressions on the OXT of each timepoint separately, including the self-reported satiety and appetite as co-variates, the group difference remained for the timepoint after lunch and after the study visit, while appetite by itself had a significant main effect on the OXT levels after waking and after the study visit, and satiety after lunch (**Table S2**).

| **Table S1**: Oxytocin across Timepoint, controlled for Appetite and Satiety | | | | |
| --- | --- | --- | --- | --- |
| Timepoint | Group | Appetite | Satiety | F statistics |
| Waking | β = 0.63 p = 0.383 | **β = -0.76** **p = 0.040*** | β = -0.67 p = 0.078 | **F(3,82) = 1.88, p = 0.030*** |
| Lunch | **β = 2.86** **p = 0.006**** | β = 0.01 p = 0.984 | **β = -1.28** **p = 0.033*** | **F(3,82) = 4.38, p = 0.007**** |
| Pre Task | β = 2.08 p = 0.072 | β = -0.58 p = 0.537 | β = -0.26 p = 0.740 | F(3,82) = 1.13, p = 0.342 |
| Post Task | **β = 1.86** **p = 0.035*** | **β = -1.10** **p = 0.040*** | β = -0.79 p = 0.138 | F(3,82) = 2.65, p = 0.052 |

Note: The values here demonstrated represent p-values of main effects in the four separate linear regressions.

**Insecure Attachment; separate per dimension**

We ran linear regressions on the ECR-R subscores separately, providing distinct statistics for each of the dimensions, Figure S1.


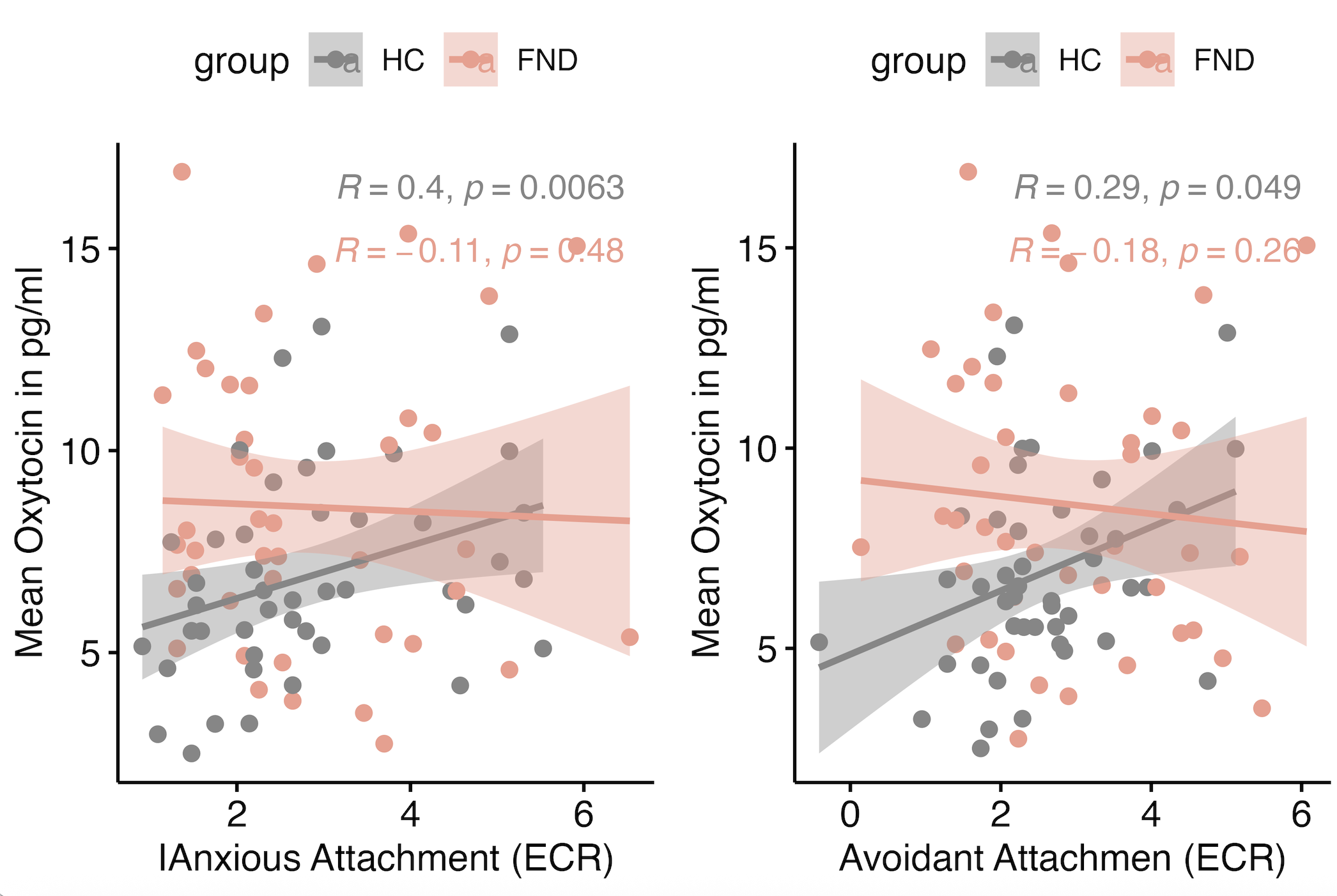


Figure 1: **Association between attachment dimensions and salivary oxytocin, separated per group.**
(A) Shows the dimension of Anxious attachment and (B) shows the dimension of Avoidant attachment in interaction with salivary oxytocin (pg/mL). Each point represents one participant; colors denote groups. For each group, an ordinary least-squares regression line is shown with its shaded 95% confidence band. Group-specific Spearman rank correlations (ρ) and p-values are overprinted on the panels (computed within group).
 ECR-R = Experiences in Close Relationships; pg/mL = picograms per milliliter.
